# Interaction Study of an Amorphous Solid Dispersion of Cyclosporin A in Poly-Alpha-Cyclodextrin with Model Membranes by ^1^H-, ^2^H-, ^31^P-NMR and Electron Spin Resonance

**DOI:** 10.1155/2014/575719

**Published:** 2014-05-05

**Authors:** Jean-Claude Debouzy, David Crouzier, Fréderic Bourbon, Malika Lahiani-Skiba, Mohamed Skiba

**Affiliations:** ^1^IRBA, RNI-Biophysics Laboratory, 24, avenue des Maquis du Grésivaudan, BP 82, 38702 La Tronche Cedex, France; ^2^Galenic Pharmaceutical Laboratory, LAGEP UMR CNRS 5007, UFR Medicine and Pharmacy, 22 boulevard Gambetta, 76183 Rouen, France

## Abstract

The properties of an amorphous solid dispersion of cyclosporine A (ASD) prepared with the copolymer alpha cyclodextrin (POLYA) and cyclosporine A (CYSP) were investigated by ^1^H-NMR in solution and its membrane interactions were studied by ^1^H-NMR in small unilamellar vesicles and by ^31^P ^2^H NMR in phospholipidic dispersions of DMPC (dimyristoylphosphatidylcholine) in comparison with those of POLYA and CYSP alone. ^1^H-NMR chemical shift variations showed that CYSP really interacts with POLYA, with possible adduct formation, dispersion in the solid matrix of the POLYA, and also complex formation. A coarse approach to the latter mechanism was tested using the continuous variations method, indicating an apparent 1 : 1 stoichiometry. Calculations gave an apparent association constant of log Ka = 4.5. A study of the interactions with phospholipidic dispersions of DMPC showed that only limited interactions occurred at the polar head group level (^31^P). Conversely, by comparison with the expected chain rigidification induced by CYSP, POLYA induced an increase in the fluidity of the layer while ASD formation led to these effects almost being overcome at 298 K. At higher temperature, while the effect of CYSP seems to vanish, a resulting global increase in chain fluidity was found in the presence of ASD.

## 1. Introduction

One challenging task in the manufacturing process is to improve the bioavailability of poorly water-soluble drugs. Thus, in recent decades, numerous potentially bioactive pharmaceutical ingredients (APIs) were found to have only low aqueous solubility. As a result, oral delivery of poorly water-soluble drugs often results in low bioavailability. Poorly water-soluble drugs cannot achieve dissolution and therefore have great difficulty passing through digestive fluid to contact absorbing mucosa and be absorbed. If the drug molecules' dissolution process is slow, due to inherent physicochemical properties of the molecules or formulation factors, then dissolution may be the rate-limiting step in absorption and will influence drug bioavailability. This is the case with class II drugs, for example, cyclosporine A (CYSP) (according to the drug Biopharmaceutics Classification System (BCS)). Cyclosporin A (CYSP), a hydrophobic cyclic peptide, is widely used as an immunosuppressant drug for transplant therapy [1, 2]. For this specific kind of drug, many enabling technologies are available for the formulator to consider, including lipids, cosolvents, surfactants, nanoparticles, cyclodextrin complexes, and amorphous solid dispersions. The suitability of a particular formulation approach depends largely on the physicochemical properties of the active pharmaceutical ingredient (API) [3]. Among these methods, the preparation of amorphous solid dispersions (ASD) with cyclodextrin copolymer (POLYA) is particularly attractive for many poorly water-soluble drug candidates [4] because these formulations offer many of the advantages of more conventional solid oral dosage forms and also provide faster dissolution rates and higher drug concentrations in the gastrointestinal milieu [3]. However, its limitation is its toxicity [5]. Among several mechanistic hypotheses, several studies addressed possible interactions of CYSP with biological membranes. The first ESR studies of CYSP's interactions with model membranes failed to identify any dynamics or structural consequences resulting from the presence of CYSP [6]. By way of contrast, small-angle X-ray diffraction and differential scanning calorimetry (DSC) studies of the effect of CYSP's interactions with model membranes composed of dimyristoylphosphatidylcholine (DMPC) bilayers showed that CYSP affected the fatty acyl chains in the bilayer, especially the part of the chain proximal to the head group [7]. These results were in good agreement with other more recent works performed on different phospholipid (dipalmitoylphosphatidylcholine (DPPC)) bilayers using other spectroscopic methods (^2^H-NMR) [8]. The goal of the present paper was to investigate the membrane interactions of this ASD in comparison with POLYA and previous studies on CYSP. As a first step, the stoichiometry and apparent constant affinity were estimated; then, its interactions with membranes were investigated using synthetic membranes in combination with ^31^P- [9, 10], ^2^H-NMR [11, 12] and ESR methods.

## 2. Materials and Methods

### 2.1. Materials

**Figure 1:**
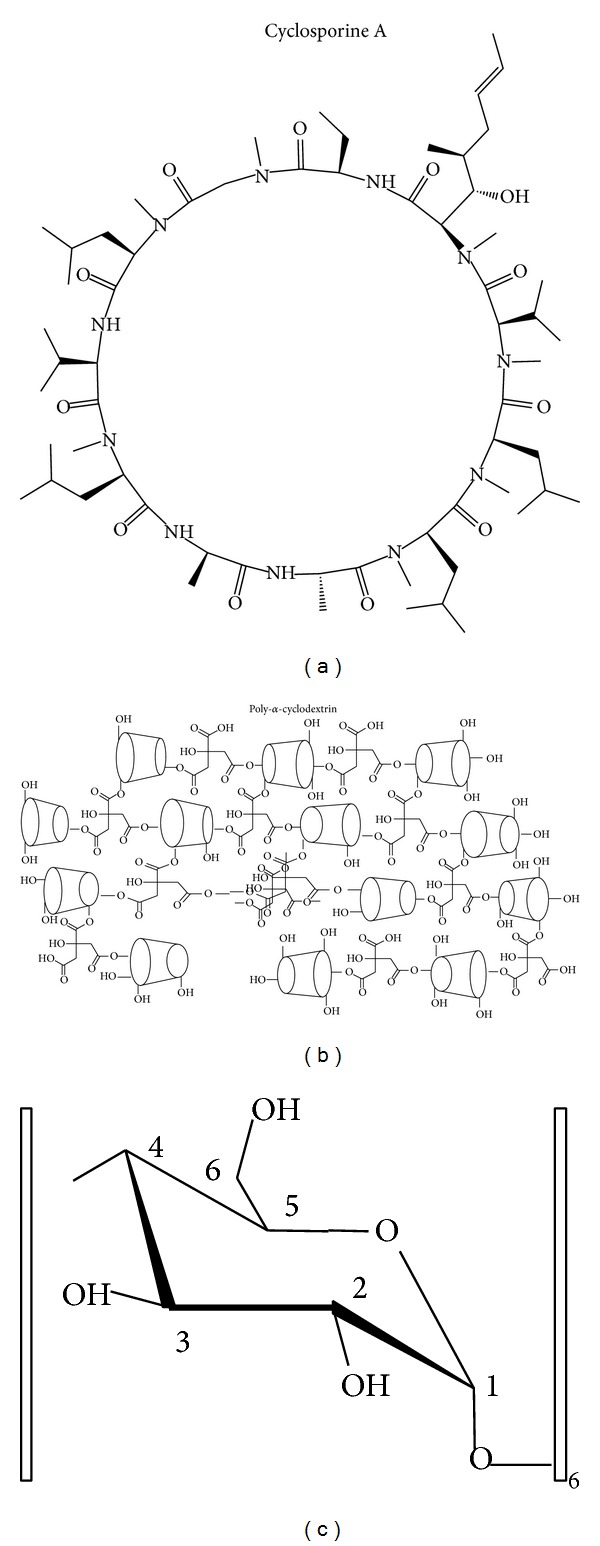
Cyclosporine A (CYSP) and poly-alpha-cyclodextrin copolymer (POLYA) structures with the proton nomenclature of the CYSP glucose building block used in the NMR experiments.

All salts and phospholipids (dimyristoylphosphatidylcholine (DMPC), egg yolk lecithin (EPC), and phosphatidic acid) were purchased from Sigma (La Verpillière, France) and used as received. Deuterated solvents and deuterium-depleted water were from Eurisotop (91191, Saint-Aubin, France). Chain perdeuterated DMPC (DMPC-d_54_) was from Interchim (Montluçon, France). DOXYL-stearic acid (5NS) for ESR experiments was from SIGMA-Aldrich. Crystalline cyclosporine (extra pure) was received from Poli, Italy (CYSP, Figure 1(a)). Purity was checked by recording ^1^H-^13^C NMR spectra and in standard correlation spectroscopy experiments (COSY, HMQC) [13] in DMSO. Copolymer alpha-cyclodextrin (POLYA) was synthesized as described in [14, 15] and an amorphous solid dispersion (ASD) of cyclosporine was prepared using the spray-dried dispersion method described by Boukhris et al. [4]. The purity was checked as described in the published studies (an average molecular weight of 240,000 was assumed, with a polydispersity index of 8 (see Figure 1(b)) [15].

### 2.2. Model Membranes

Multibilayers (MLV): DMPC liposomes for ^31^P experiments were prepared by successive freeze/thaw cycles (5) until a homogenous milky sample was obtained [10]. The suspensions were degassed under nitrogen gas and then introduced into NMR tubes and sealed. The final lipid concentration was 50 mM, while CYSP/DMPC in mixed systems was 6% M/M as described in previous studies. Various W/W proportions of DMPC to POLYA (from 3 to 12) and POLYA-CYSP complexes (from 3 to 15) were tested. The results presented here used 4/50 complexes to DMPC and 3/50 POLYA to DMPC weight ratios.

The same procedures were used to prepare multilayers for ^2^H-NMR experiments, except that 25% DMPC with perdeuterated chains was used (DMPC-d_54_) to prepare the liposomes.

### 2.3. Methods

#### 2.3.1. NMR Experiments

^1^H-NMR experiments were recorded at 295 K on a Brüker AVANCE III-400 spectrometer using a presaturation of the water resonance and a spectral width of 10 ppm. As preliminary relaxation studies evoked T1 values around 0.6 s, a recycling delay of 2.5 s between pulses was used with *π*/3 pulses (4.8 *μ*s). The chemical shifts were referenced by setting the water resonance at 4.75 ppm. ^1^H-NMR attribution was considered in reference to natural alpha-cyclodextrin and controlled by standard correlation spectroscopy experiments [13].

The first recordings of the POLYA/CYSP complex showed chemical shift variations with respect to POLYA, suggesting that a molecular association operating under fast exchange kinetics conditions was present. Using its very coarse approximation of a complex formation, the classical method described by Job [16–18] was used to extract an apparent macroscopic stoichiometry of the complex, while the SIMPLEX mathematic determination method (EXPREX or MURIEL-X algorithms generously provided by Bruno Perly, CEA Saclay, France) gave estimations of the apparent association constant [19].

^31^P-NMR experiments were performed at 162 MHz. Phosphorus spectra were recorded using a dipolar echo sequence (*π*/2-*t*-*π*-*t*) with a* t* value of 12 *μ*sec, a recycling delay of 2.5 s, and a composite proton decoupling. Phosphoric acid (85%) was used as external reference.

^2^H-NMR experiments were performed at 61 MHz. Deuterium spectra were recorded using a quadrupolar echo sequence (*π*/2-*t*-*π*/2-*t*) with a* t* value of 15 *μ*sec and a 10 s recycling delay. The free induction decay was shifted in fractions of the dwelling time to ensure that the effective time for the Fourier transform corresponded to the top of the echo [20]. The sample temperature was regulated within 1°C by a BVT-1000 unit.

^2^H-NMR spectra treatment: in order to extract suitable quadrupolar splitting measurements (Δ*ν*_*Q*_), the spectra were de-Paked according to the Seelig procedure [21]. This allowed a fluidity profile to be built and calculation of the carbon-deuterium bond segmental order parameter *S*_CD_ using the following classical relation [11, 20]:

(1) $$\begin{array}{c} S_{\text{CD}}=\frac{3\cos^{2}\beta -1}{2}, \end{array}$$

where *β* is the average angle between the carbon deuterium bond and the direction perpendicular to the bilayer normal. This value can be drawn for a given CD bond from a measurement of the quadrupolar splitting, Δ*ν*_*Q*_ (kHz), that is, the frequency separation of the two deuterium resonances on the spectrum, by using the following relation:

(2) $$\begin{array}{c} \Delta \nu_{Q}=\left(\frac{3}{4}\right)\left(\frac{e^{2}qQ}{h}\right)S_{\text{CD}}, \end{array}$$

where (*e* ^2^ *qQ*/*h*) is the quadrupolar coupling constant, equal to 170 kHz for aliphatic carbon deuterium bonds [22].

### 2.4. ESR Experiments

The DMPC dispersions were prepared as for ^31^P-NMR experiments. Each 100 *μ*L sample of this suspension (with or without CYSP, POYA, or ASDP) was then labeled with 2 *μ*L of a radical nitroxide marked probe solution (10^-2 ^M in dimethylsulfoxide); the probe was 5 DOXYL-stearic acid (5NS). After labeling, the sample was transferred by capillary action into a 20 *μ*L Pyrex capillary tube and incubated for 10 minutes. These tubes were placed in a 3 mm diameter quartz holder and inserted into the cavity of a Bruker ESP 380 spectrometer (Karlsruhe, Germany) operating at 9.79 GHz. Complete membrane incorporation of the spin labels was ascertained by the absence in the spectra of highly resolved EPR lines corresponding to free rotating markers. The spectra were recorded at temperatures below (292 K), around (297 K), and over (308 K) the temperature transition under the following conditions: microwave power 20 mW, modulation frequency 100 kHz, modulation amplitude 2.868 G, and time constant 327 msec. The parameters measured were the hyperfine splitting constants (2*T* ^//^ and 2*T* ^⊥^), allowing for calculation of the order parameter [23]:

(3) $$\begin{array}{c} S=\frac{1.723\left({2T}^{//}-2T^{⊥}-C\right)}{\left(T^{//}+2T^{⊥}+C\right)} \end{array}$$

with *C* = 1.4 − 0.053∗(*T* ^//^ − *T* ^⊥^). 2*T* ^//^ is related to the molecular organization surrounding the probe and accounts for an order parameter. If 2*T* ^//^ increases, then the order increases at this level of the membrane, that is, the outer hydrophilic moiety of the layer.

## 3. Results

### 3.1. Characterization of Amorphous Solid Dispersion (ASD)

**Figure 2:**
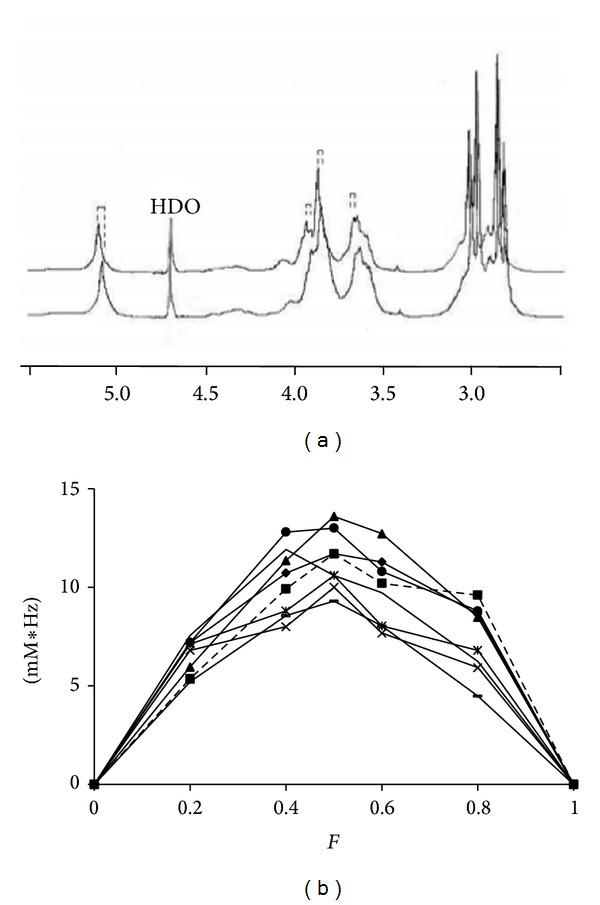
(a) ^1^H-NMR spectra (297 K, D_2_O) of pure 2 mM POLYA (bottom trace) and the 1/1 preparation (top trace); dashed lines represent several of the chemical shift variations observed. (b) Job plots built from the different proton chemical shift variations: 5.29 ppm (d) H1** —**; 3.65(t) H2** ▲**; 4,05(t) H3 ∗; 3.95(m), H4 ×; 4.27(m), H5 ●; 3.00(m), (H6-6′) ■.

ASD was prepared by the classical slow evaporation [16] method for a total concentration of 2 mM, with the POLYA/CYSP molar ratio scaled from 1/9 to 9/1 M/M. The ^1^H-NMR spectrum of POLYA (D_2_0, 297 K) is presented as the bottom trace of Figure 2(a). As described previously [15, 24], the method of synthesizing the POLYA yields polymers of alpha cyclodextrin connected by citric acid building blocks [25], with a mean molecular mass of 240,000 and a polydispersion index of 8. This means that, in addition to the main macromolecular assembly, smaller objects are also present, even if in small amounts [4]. The corresponding ^1^H-NMR spectrum thus consists of relatively broad lines (6 Hz) that could be assigned by comparison with natural alpha-cyclodextrin and/or by recording standard basic COSY experiments [13, 14]: 5.29 ppm(d), H1; 3.85(t)H2; 4.43(t)H3; 4.27(m), H4; 4.05(m), H5; 4.3(m)(H6-6′).

In the coarse study of the association between CYSP and POLYA, the POLYA resonances were considered as a whole while a CYSP molecular mass of 2000 was assumed. From this, different apparent molecular ratios of *R* = CYSP/POLYA were prepared using the slow evaporation method of complexation described classically [11], with the total concentration kept constant at 2 mM. The result is presented as the top trace of Figure 2(a). As shown in the graph, the presence of cyclosporin results in chemical shift variations in POLYA resonances, indicating rapid exchange kinetics. These downfield ^1^H chemical shift variations were observed with increasing *R*. Since no clear conclusion could be drawn from Higuchi solubility diagrams, we attempted to plot in a Job-plot manner the weighted chemical shift variations in these resonances as a function of the molar fraction of POLYA. In the case of inclusion complex formation, such plots allow a maximum to be observed for the fraction corresponding to the stoichiometry (Job-plot method) [17, 24] (Figure 2(b)). Such a maximum was observed on all traces for *F* = 0.5, suggesting an apparent 1 : 1 stoichiometry of this association (even if the asymmetrical shape of the curves and the variations observed in all resonances run counter to a simple inclusion). Despite its probable meaninglessness, an apparent constant Ka was calculated mathematically [25], giving a coarse estimation of log Ka ≈4.2–4.8 M^−1^.

This led us to select a 1/1 preparation for the following experiments using the spray-dried dispersion method.

As, on the one hand, POLYA was supposed to enhance the biodisponibility of CYSP and, on the other, the interactions of water insoluble CYSP with membranes had been investigated in previous studies, it was of interest to explore such interactions of POLYA and especially of the POLYA/CYSP complex itself with membranes. This study is proposed in the next section.

### 3.2. Interactions with Membranes

Homogeneously prepared systems consisting of synthetic phospholipid dispersions (MLV) offer a suitable tool with which both structural and dynamic consequences of drug-membrane interactions are observed. The results are presented in this section, using ^31^P- and ^2^H-NMR spectroscopy and ESR spectroscopy on CYSP, POLYA, and a 1/1 complex (ASD) containing MLV of DMPC.

### 3.3. Membrane Dynamics Study by ^31^P-^2^H-NMR and ESR

#### 3.3.1. The Polar Head Group Level: ^31^P-NMR Experiments

**Figure 3:**
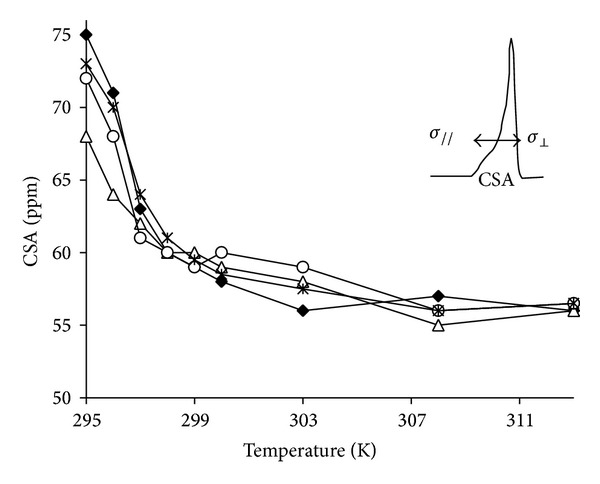
^31^P-NMR of pure DMPC (50 mM concentration), with weighted chemical shift variations in POLYA protons as a function of the POLYA molar fraction,** ◆**; or containing 4 mM CYSP ∆; 4 mg POLYA ○; or 4 mg ASD ∗; insert shows a typical spectrum of DMPC dispersion at 298 K. Arrows represent the chemical shift anisotropy.

As shown in the insert in Figure 3, the ^31^P-NMR spectrum of the pure DMPC dispersion (MLV) was typical of an axially symmetric powder pattern, with a chemical shift anisotropy of 58 ppm typical of DMPC bilayers in their liquid crystalline phase (298 K) [26]. The chemical shift difference between the lowfield and highfield edges of the ^31^P-NMR spectrum is called the chemical shift anisotropy (CSA, ppm) and is directly related to fluidity reorientation at the polar head level where the phosphorus nuclei are located. Hence, a mobile phosphorus group gives a single narrow resonance (several Hz) as detected in a true solution or with small structures (micelles), while solid state phosphorus gives extremely broad contributions (greater than 100 ppm). Note that membrane fluidity increases (and CSA decreases) with temperature, with a special jump at the transition temperature between the gel phase and the liquid crystal structure (around 297 K for DMPC). Thus, the plot of CSA as a function of temperature provides a good overview of the membrane dynamics at the polar head level where the phosphorus nuclei are located. Such plots are presented on the trace in Figure 3 for pure DMPC dispersions and for MLV containing CYSP, POLYA, and the 1 : 1 complex (ASD).

**Figure 4:**
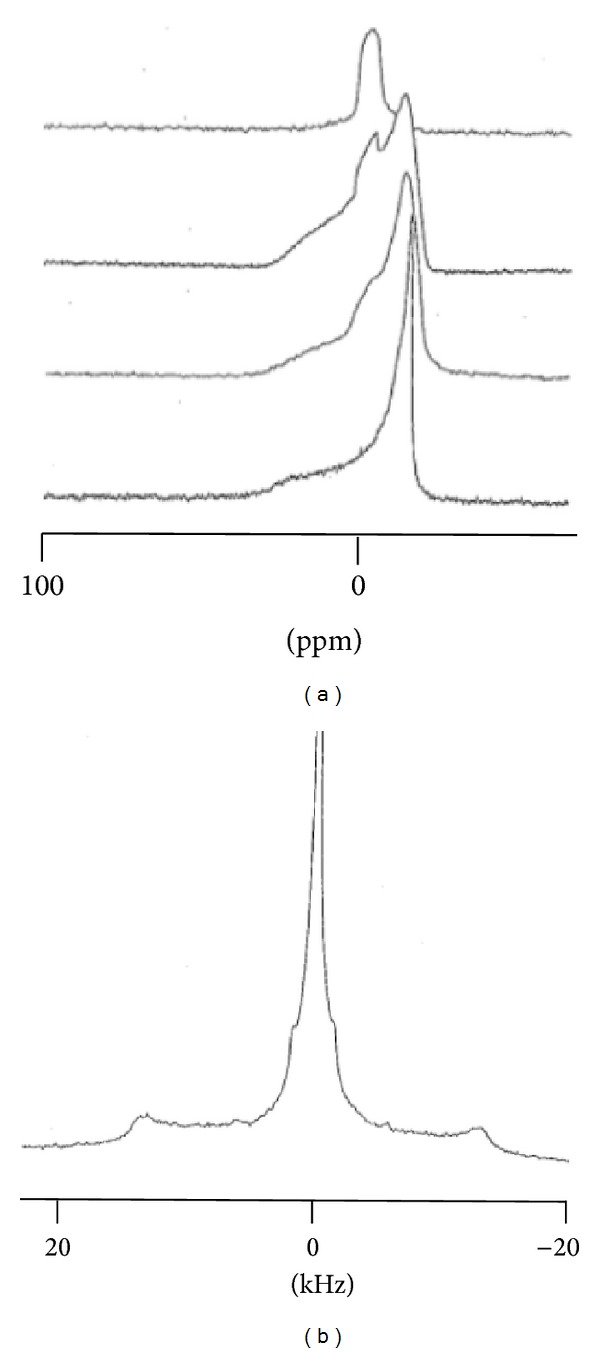
(a) Time evolution of ^31^P-NMR spectra of POLYA containing MLV (6/50 W/W). Proceeding from bottom to top, each spectrum was recorded 6 hours after the one below it. (b) The corresponding ^2^H-spectrum recorded after the top trace in Figure 4(a).

As expected, a decrease in CSA (of around 18 ppm) was observed between the low (295 K) and high temperatures (313 K), with a transition-related jump at around 297 K. Such a temperature dependence was also found for CYSP, POLYA, and ASD containing MLV. However, in the case of the CYSP-containing system, the transition temperature was slightly lower (up to 1 K), while its amplitude was lowered by 10 ppm, in agreement with an interaction with the polar head group, even of relatively weak importance, possibly being related to an enhanced fluidity below the transition temperature. In addition, the curves built with POLYA and ASD were very similar and close to that constructed with DMPC alone, with the same transition temperature and only a limited reduction in CSA at low temperature, indicating only minor interactions at the polar head level at the concentration used. Moreover, no isotropic contribution was found in the spectrum, precluding any solubilization or detergent effect. However, by using higher POLYA/DMPC or ASD/DMPC weight ratios, *R*, a broad isotropic component was detected immediately for *R* = 1/5 or, following some passage of time, when *R*exceeded 6/50 (see Figure 4(a)). Due to its 600 Hz linewidth, such a structure had to be distinguished from a solubilization, which should provide a resolved line of couple of tenth Hz wide at the same position, corresponding rather to membrane destruction into smaller heterogeneous fragments. This point is supported by the line shape of the corresponding ^2^H-NMR spectrum (Figure 4(b)). Hence, even if a strong isotropic line is detected at the isotropic position (with a line width of 1 kHz) residual doublets (of 6, 10, and 24 kHz) still remain observable, revealing that some structure (membrane fragments, etc.) is present.

Nevertheless, this feature cannot be explained at this point. Spin labeling and the ESR method were therefore used to observe the membrane chain sides close to the polar head group.

#### 3.3.2. The Acyl Chain Level Close to the Polar Head: ESR 5NS Experiments

**Figure 5:**
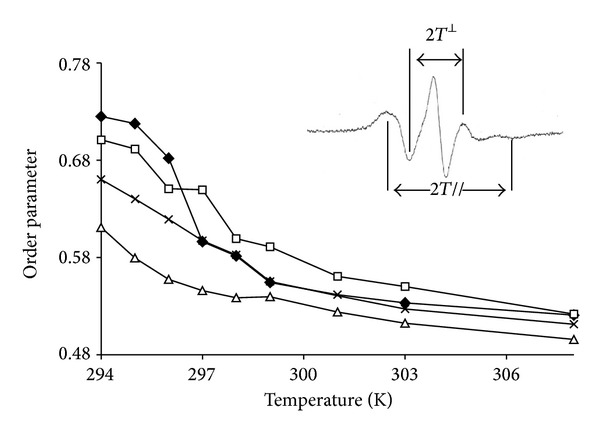
ESR-5NS experiments: plot of the apparent order parameter *S* as a function of temperature for pure MLV of DMPC** ◆** and in the presence of 4 mg CYSP (□), 4 mg POLYA (∆), and 4 mg ASD (×). The insert shows the spectrum parameters used to calculate *S*.

As described in Section 2.3, an estimate of order parameter can be extracted from ESR spectra (measurements of hyperfine splittings 2*T* ^//^ and 2*T* ^⊥^ **)**, as shown in the inset of Figure 5. This allows the temperature dependence to be observed as well as the transition temperature just below the surface, at the carbon 5 level where the spin label is grafted on the stearic acid. The typical trace and transition temperature (297 K) were found for DMPC. This was also roughly the case for CYSP-containing systems; however, even though the transition is respected, one can note that overall order is increased above this temperature while, conversely, fluidity is increased below this point. The result is a smoothing of the transition. When POLYA is present, a transition is no longer clearly observable and a loss of local order is apparent over the whole temperature range, consistent with fluidization at this level. The curves built from ASD-containing systems demonstrate an intermediate situation, that is, a recovery of the transition—even if smoothed—with an intermediate fluidity profile between the POLYA system on the one hand and DMPC or CYSP-containing MLV on the other.

At this stage these different aspects appear homogenous with the phosphorus results, even if more markedly observed in the present case. From this, it was of interest to perform further investigations at deeper levels of the layer, that is, the whole acyl chain, which were realized by recording ^2^H-NMR spectra of chain perdeuterated DMPC (DMPD) under the same conditions.

#### 3.3.3. The Overall Acyl Chain Level: ^2^H-NMR

**Figure 6:**
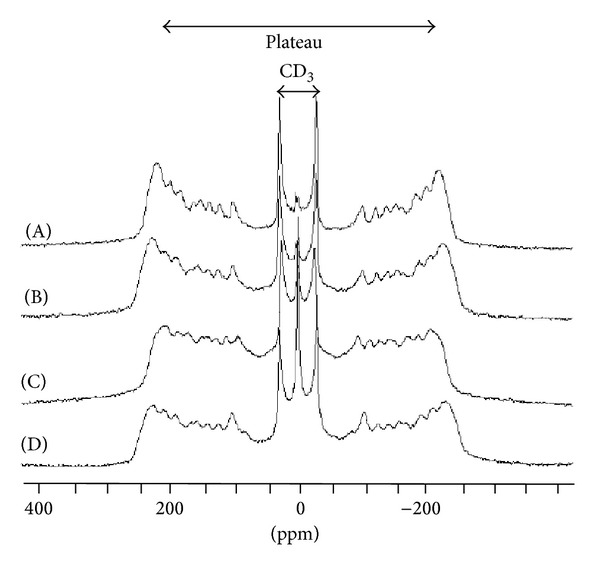
(A) ^2^H-spectrum of pure DMPD-d_54_ in H_2_O at 298 K and in the presence of 4 mg CYSP (B), 4 mg POLYA (C), and 4 mg ASD. The dashed line shows the shifts in the plateau resonance.

Figure 6(A) shows the spectrum of a pure DMPC-d_54_ (dimyristoylphosphatidylcholine with perdeuterated chains) dispersion. This spectrum istypical of phospholipid bilayers in the liquid crystal phase (temperature of 298 K). Such a spectrum appears as a superimposition of symmetrical doublets, each doublet corresponding to a CD_2_ group of the acyl chain; thus, for a given doublet, the splitting of (Δ*ν*_*Q*_) is directly related to the local chain fluidity (see Section 2.3). This splitting can be used in a first approximation as an order parameter. As the acyl chain fluidity decreases from the terminal methyl group (CD_3_) to the methylenic groups close to the polar head of the lipids (the so-called “plateau region,” from C-2 to C-8), the resulting spectrum consists of (i) an inner doublet with a quadrupolar splitting of 4 kHz attributed to the CD_3_ group, (ii) doublets with increasing quadrupolar splitting assigned to successive CD_2_ groups from C14 to C9, and (iii) an external edge doublet, attributed to the deuterium in the C2–C8 plateau region where a 29 kHz quadrupolar splitting is measured. In the presence of CYSP (Figure 6(B)), where the overall trace looks very similar, one can notice an increase in quadrupolar splitting at the plateau region level (31 kHz) and also all along the different doublets; while smaller and smaller as one moves along the chain down to the CD_3_ doublet, this difference is almost negligible (4.2 kHz).

The situation is quite different when POLYA (*R* = 1/5) is present in the MLV; here, an = homogenous diminution in quadrupolar splitting is observed for all resonances (e.g., from 4 to 3.6 kHz for the CD_3_ doublet and from 29 to 26.6 kHz for the plateau contribution), indicating overall fluidization of the bilayer at 298 K (Figure 6(C)). In addition, the use of a preformed complex in MLV (*R* = 1/5), while almost restoring the splitting at the plateau level (28 kHz), induced an increase in CD_3_ splitting (to 4.4 kHz), as shown in Figure 6(D).

**Figure 7:**
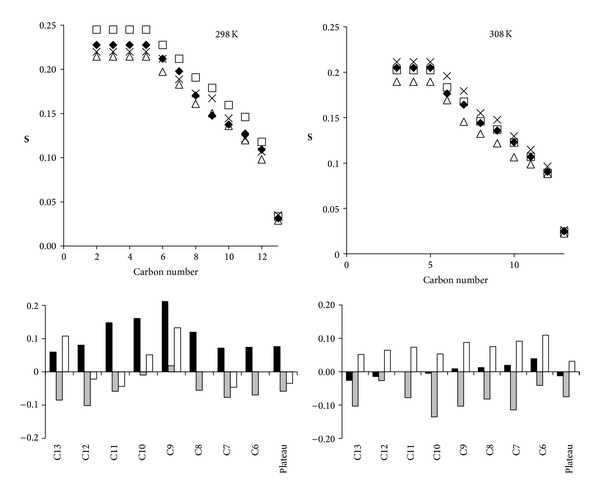
^2^H-NMR left column: fluidity profile (order parameters) plotted at 298 K for DMPC-*d*_54_ alone** ◆** and in the presence of 4 mg CYSP (□), 4 mg POLYA (∆), and 4 mg ASD (×). The numbering refers to the number of the carbon on the acyl chain.

These observations are also visible in the fluidity profile shown in Figure 7. The data used to obtain the top traces were also used to build, for all CD groups, histograms of relative local fluidity variation by plotting for each resonance in a given system X:

(4) $$\begin{array}{c} R=\frac{\text{Q}{\text{S}}_{\text{X}}-\text{Q}{\text{S}}_{\text{DMPC}}}{\text{Q}{\text{S}}_{\text{DMPC}}}, \end{array}$$

where QS_X_ is the quadrupolar splitting of the system X and QS_DMPC_ that of the corresponding resonance in the DMPC reference MLV (bottom histograms of Figure 7). Such a plot, while confirming the previous results, also shows that the most significant rigidification induced by CYSP takes place in the middle of the chain, even if it is also close to the carbonyl group in the plateau region. Similarly, the fluidizing properties of POLYA appear to be present at both ends of the chain, while the presence of the complex almost overcomes the effects of CYSP. Temperature dependence: as mentioned in the previous section, the dynamics of DMPD multilayers are characterized by a phase transition from a gel state to a liquid crystal state at a given temperature. This specific transition temperature in DMPD-d_54_ is also 297 K, with a dramatic reduction in quadrupolar splitting (QS) noted around 297 K. This transition temperature was not significantly modified between the different samples used (not shown). However, by increasing the temperature, besides the expected reduction in the QS values (reflecting an increase in fluidity), the fluidity profiles and relative local fluidity modifications appear quite different (e.g., see Figure 7 in the right column at 308 K). With regard to DMPD, the CYSP effects appear nearly negligible, while the fluidizing effect of POLYA was more pronounced and homogenous. Furthermore, the presence of the complex results in an overall homogenous rigidification at all chain levels.

## 4. Discussion

The goal of the present paper was to investigate the interactions of a preformed cyclosporine complex with a copolymer of alpha-cyclodextrin (ASD) and to study its interactions with membranes by comparison with those of CYSP and POLYA alone. Due to the polydispersity of POLYA (DI of 8), the first step was to select the experimental conditions, that is, the concentration and complex stoichiometry of ASD, to use when in the presence of membranes.

Previous studies [27] evoked different mechanisms of interactions, either a true inclusion of CYSP in the cyclodextrin cavity as suggested by ^13^C-NMR HRMAS spectra recording, or solid dispersion in the POLYA matrix, favored by the SDD mode of preparation. The same author also mentioned [27] that such a dissolution was thought to be related to the absence of crystallinity and improved wettability of CYSP A. Another possible mechanism would be a solubilization of CYSP by an interaction with POLYA without any inclusion, mediated by hydrogen bonds. After recording several preliminary ^1^H-NMR spectra of POLY CYSP under different W/M ratios and concentrations, and as chemical shift differences were noted in the POLY resonances, we decided to use complexation studies methods on this model. Higuchi and Connors [28] solubility diagrams did not give clear information (rapid steady state, no ambiguous curvature of the line slope), so we decided to use the continuous variations method [17]. After constructing such a Job plot, it in fact proved unrealistic to propose a stoichiometry as well, due to polydispersion of the POLYA. It is unlikely that the interactions of CYSP A with POLYA, either in oligomeric assemblies or with a 30,000 MW assembly, would be similar. This coarse approach did not lead to an acute determination of the stoichiometry or affinity constant; however, this was proposed considering the following.

- The contribution of small MW entities (oligomers) is almost always very limited, as shown in other papers published on the synthesis and characterization of POLYA [27]. The contribution of this component cannot be distinguished by comparison with the broad resonance of the macromolecular structures.
- Heterogeneous/randomly substituted cyclodextrin complexation studies have been performed in the past (e.g., poly randomly methylated cyclodextrins, RAMEB) and published [29, 30]. Conversely, natural products such as natural phospholipids—which are always mixtures of various chain lengths and degrees of unsaturation—have been investigated in the presence of cyclodextrins [19].
- The historical Job paper [17] was designed to build such plots by using any observable variable, which may mean fluorescence frequency, absorbance, DSC or IR band, or, as here, chemical shift variations.

Nevertheless, although the maxima of all traces were close to *F* = 0.5 (apparent stoichiometry of 1), with calculations giving an apparent association constant of 4.5, it cannot be assumed that inclusion in the cyclodextrin cavity is the exclusive mechanism. Hence, such a mechanism would have given mainly or exclusively chemical shift variations in the H3 and H5 resonances, located well inside the torus structure. Conversely, simple adducts would have modified external proton resonances. As assumed by Boukhris et al. [4], different mechanisms would in fact be present. However, the macroscopic result led us to use 1/1 preparations of CYSP/POLYA by SDD for membrane studies. Preliminary ^1^H-NMR experiments in small unilamellar vesicles (SUV) of lecithins using classical paramagnetic broadening methods (not shown) [31, 32] had shown that all three species truly interacted with membranes and these interactions were probably not at the level of the choline groups. These results were not in agreement with older works performed by ESR in large unilamellar vesicles of DMPC [5], where no significant interaction was found. This discrepancy led us to study these interactions further by using a membrane system more adapted to structural and dynamic studies, that is, MLV of DMPC, in combination with static solid NMR technics. As a control the experiments described by Stuhne-Sekalec and Stanacev [6] were also replicated.

CYSP interactions with membranes had been suspected early on and were investigated from the late 90s onward [8]. According to these studies, ^31^P-NMR in MLV confirmed that the overall interactions of CYSP, POLYA, or ASD with the phosphorus in the head group were weak, except when high concentrations of POLYA were present. Membrane damage was then identified, suggesting a limit to the amount of POLYA which is reasonable to use (molar ratios exceeding 6/50). ESR results also confirmed a limited smoothing and lowering of the transition in the presence of CYSP, in agreement with a superficial interaction with the polar head. The result is an increased fluidity at low temperature and rigidification above the transition temperature. It is noteworthy that such a feature is not observed in the presence of a preformed complex. At the concentration used, fluidizing properties of POLYA are not apparent and cannot overcome CYSP-induced rigidification; a geometric hindrance appears to be the most probable hypothesis. In addition, a competition for CYSP between the membrane and POLYA also has to be considered.

Looking at the chain level in the membrane, CYSP was found to increase the order parameter all along the chain (^2^H-NMR), especially close to C10 at 298 K, but of limited amplitude in the plateau region [11]. A previous study of Wiedmann et al. [8] used dipalmitoylphosphatidylcholine; a longer chain length would modify the mutual relationships between the chain and CYSP. Similarly, they detected only minor effects at the polar head group level where the phosphorus is located. This does not run counter to the broadening of the chemical shift anisotropy previously observed in the presence of ethanolamine phospholipids [21], suggesting that the nature of the polar head group would also play a role in the interactions.

Conversely, POLYA exhibits increased fluidity all along the chain; hence, POLYA was designed to be soluble and able to solubilize hydrophobic molecules; as confirmed by its amphiphilic properties and good solubility in water (1 mg/mL), these properties are all in favor of interactions with membranes. As shown in Figure 7, such an increase in fluidity appears to be sufficient to overcome CYSP-induced rigidification when the complex is formed. The effects are also present below and especially close to the transition temperature (at 298 K, present in Figure 7). When the temperature rises (308 K, see Figure 7) closer to biological conditions, the membrane interactions of CYSP almost completely vanish, while POLYA- and ASD-induced fluidization appear to become more effective. If it is considered that only the OH of the hydrophobic molecule CYSP is appended as a lateral group (MeBMt-1) to the main ring structure, then the molecule can both be embedded in the layer and form a hydrogen bond close to the carboxylic group of the chains, in agreement with very limited interactions at the polar head level. This is also supported by several papers [33, 34] that consider CYSP as being loaded in the membrane interior with the MeBmt-1 amino acid folded over the molecule itself assuming a globular shape. Any fluidizing reagent (POLYA), temperature jump, or hiding of this hydroxyl via complex formation would minimize CYSP-chain interactions, in accordance with the data recorded at 308 K.

## 5. Conclusions

Finally, this work shows that POLYA can truly solubilize CYSP: this is probably achieved by forming a complex. The dispersion of hydration water in POMR experiments on the different systems would also probably show the role of wettability in such interactions. In addition, POLYA interacts with membranes, directly by fluidizing effects at the chain level (especially at biological temperatures) and by overcoming the rigidifying effect of CYSP just over the transition temperature of DMPC. Discrepancies with some published studies still remain, such as the precise location of CYSP, ASD, and POLYA interactions with the membranes. This will require studying different head groups and also chain lengths. These conclusions also have to be validated in biological models (e.g., in red blood cells using ESR methods) and finally in terms of biocompatibility to identify the mechanism of the membrane damage that occurs at high POLYA or ASD concentrations. These experiments are now in progress.
